# A theoretical mathematical model for assessing diclofenac release from chitosan-based formulations

**DOI:** 10.1080/10717544.2020.1797242

**Published:** 2020-07-28

**Authors:** Manuela Maria Iftime, Daniel Lucian Dobreci, Stefan Andrei Irimiciuc, Maricel Agop, Tudor Petrescu, Bogdan Doroftei

**Affiliations:** a Petru Poni Institute of Macromolecular Chemistry, Romanian Academy of Sciences, Iasi, Romania; b Department of Physical and Occupational Therapy, Vasile Alecsandri University of Bacau, Bacău, Romania; c Plasma and Radiation Physics, National Institute for Laser, Bucharest, Romania; d Department of Structural Mechanics, “Gh. Asachi” Technical University of Iasi, Iasi, Romania; e Department of Physics, “Gh. Asachi” Technical University of Iasi, Iasi, Romania; f Origyn Fertility Center, Clinical Hospital of Obstetrics and Gynaecology, “Grigore T. Popa” University of Medicine and Pharmacy, Iași, Romania

**Keywords:** Multifractal model, chitosan, diclofenac, drug release mechanism

## Abstract

The paper reports a new mathematical model for understanding the mechanism delivery from drug release systems. To do this, two drug release systems based on chitosan and diclofenac sodium salt as a drug model, were prepared by in situ hydrogelation in the presence of salicylaldehyde. The morphology of the systems was analyzed by scanning electron microscopy and polarized light microscopy and the drug release was *in vitro* investigated into a medium mimicking the *in vivo* environment. The drug release mechanism was firstly assessed by fitting the *in vitro* release data on five traditional mathematical model. In the context of pharmacokinetics behavioral analysis, a new mathematical procedure for describing drug release dynamics in polymer-drug complex systems was proposed. Assuming that the dynamics of polymer-drug system’s structural units take place on continuous and nondifferentiable curves (multifractal curves), it was showed that in a one-dimensional hydrodynamic formalism of multifractal variables the drug release mechanism is given through synchronous dynamics at a differentiable and non-differentiable scale resolutions.

## 1. Introduction

The rapid technological development contributed on a hand to the improvement of the human life and on the other hand to the increase of the mortal illnesses impact, such as tumors, diabetes and heart attack, and so on. The dramatic side effects of the systemic drug administration directed the research to a new domain, that of the controlled drug delivery systems, which become an important topic of our days (Tiwari et al., 2012). Basically, these systems consist in the dispersion of the drug into a matrix, which has the ability to deliver it in a controlled, prolonged manner to a targeted site. From this perspective, the development of the domain implies the investigation of new systems and of the mechanisms which governs the drug release. There are a lot of drug delivery systems explored in the recent times, but there are still certain trials that must be addressed for a successful delivery of the drugs to target site (Patra et al., 2018; Fisher et al., 2010). To be used as drug delivery systems in medical field, the corresponding matrix needs to present specific properties such as biocompatibility, biodegradability and ability to develop strong forces with the drug in order to prolong its release (Peppas & Brannon-Peppas, 2001; Craciun et al., 2019). Different materials, derived from natural or synthetic resources have been used as matrix for drug delivery systems over time (Griesser & Kambouris, 2001). By far the hydrogels, especially those obtained from natural resources, appeared to meet the requisite requirements for drugs matrix, due to the fact that besides biocompatibility, biodegradability, and lack of toxicity of the degradation by-products, they also have the advantage to mimic tissues by a proper moisture degree. Chitosan based hydrogels successfully fulfills all these required properties, being preferred for applications in medicine, but also in agriculture, water waste treatment, hygiene, food industry, and so on (Ahmadi et al., 2015; Desbrières & Guibal, 2018; Iftime et al., 2019; Abdel-Aleem & El-Aidie, 2018). The properties of the chitosan hydrogels are closely related with their preparation pathway. Those prepared by physical crosslinking are able to swell and load different drugs, but most of them have a too fast degradation rate over time (Vitaliy & Khutoryanskiy, 2015). On the other hand, those prepared by chemical cross-linking have a better stability but their degradation rate is often too low for *in vivo* applications (Peppas et al., 2000). Besides, an important issue is the chemical crosslinker, which is usually a dialdehyde with a certain degree of toxicity limiting the hydrogel bio-application (Omidian & Park, 2010). Taking account of this state of the art, our group developed a new strategy for chitosan hydrogelation with natural monoaldehydes, which are of natural origin and have no toxicity (Marin et al., 2015; Ailincai et al., 2016; Iftime et al, 2017; Marin et al., 2017; Olaru et al., 2018; Bejan et al., 2018; Iftime & Marin, 2018; Xiong et al., 2019). The hydrogels prepared by this route proved excellent ability to accommodate biologic agents, such as drugs or fertilizers (Craciun et al., 2019; Iftime et al., 2019; Ailincai et al., 2018; Iftime et al., 2020). Among these new hydrogels, that using salicylaldehyde as crosslinker lead to chitosan hydrogels which demonstrated appropriate properties for the design of drug delivery systems, such as biocompatibility, biodegradability, swelling ability, thixotropy and self-healing (Iftime et al, 2017). They proved capacity to encapsulate a model drug, diclofenac sodium salt (DCF), and further to slowly release it along 10 days, based on the strong physical forces developed between matrix and drug (Iftime et al., 2020). These excellent findings, encouraged us to further investigate these formulations in order to better understand the release mechanism, to create a proper structure-drug release correlation, and further to improve the design to reach the thorny goal of a real world application. Since the drug release is a complex phenomenon which depends by many factors, in this paper for a better understanding of the DCF release mechanism from the chitosan based hydrogels it was proposed to model the drug release kinetics by a multifractal theoretical model built by means of empiric laws and logistics type. Traditional mathematic models, such as *Zero order, First order, Korsmeyer-Peppas, Higuchi* model and *Hixson-Crowell* model were used in order to validate the multifractal model developed by us.

The above mentioned models are based on the hypothesis of homogeneity in its various forms (homogenous kinetic space, law of mass etc.) which has become almost dogmatic in classical Pharmacokinetics (PK). The functionality of such a hypothesis allowed the development of a class of differentiable models in the description of dynamics of biological systems (i.e., 'compartmental’ analysis) and mainly, of drug release dynamics in such systems. However, biological systems are nowadays understood as inherently non – differential (fractal). Specifically, the microenvironments where any drug molecules with membrane interface, metabolic enzymes or pharmacological receptors are unanimously recognized as unstirred, space – restricted, heterogeneous and geometrically fractal. It is thus necessary to define a new class of models, this time non – differentiable, in describing biological system dynamics and particularly drug release dynamics in such systems. The Fractal Pharmacokinetics implies the use of fractional calculus, expanding on the notion of dimension etc. As such, it is possible in the context of ‘compartmental analysis’ (Pereira, 2010) to describe diffusion in dense objects (Lemehaute & Crepy, 1983), dynamics in polymeric networks (Barkai & Klafter, 1998), diffusion in porous and fractal media (O’Shaughnessy & Procaccia, 1985), kinetics in viscoelastic media (Mainardi, 1994) etc. More recently, ‘compartmental analysis’ through PK allowed the modeling of processes such as drug dissolution (Kosmidis et al., 2003), absorption (Higaki et al, 2001), distribution (Karalis et al., 2003), whole disposition (Weiss, 1999), kinetics with bio – molecular reactions (Kotulskil & Weron, 1996) etc. In this work a new method for describing drug release dynamics in complex systems (evidently discarding to fractional derivative and other standard ‘procedures’ used in fractal Pharmacokinetics), considering that drug release dynamics can be described through continuous but non – differentiable curves (multifractal curves) is proposed. Then, instead of ‘working’ with a single variable described by a strict, non – differentiable function, it is possible to ‘operate’ only with approximations of these mathematical functions, obtained by averaging them on different scale resolutions. As a consequence, any variable purposed to describe drug release processes will still perform as the limit of a family of mathematical functions, this being non – differentiable for null scale resolutions and differentiable otherwise.

## 2. Materials and methods

### 2.1. Materials

Chitosan of low molecular weight (193 kDa, degree of deacetylation 82%), salicylaldehyde 98% (SA), diclofenac sodium salt (*DCF*), ethanol, glacial acetic acid, phosphate buffer (PBS) (pH = 7.4) were purchased from Aldrich and used as received.

### 2.2. Preparation of the drug delivery systems

The drug delivery systems were prepared by *in situ* hydrogelation of chitosan with salicylaldehyde in the presence of ***DCF,*** according to a procedure developed in our group (Craciun et al., 2019; Ailincai et al., 2018; Iftime et al., 2020). Using two different molar ratio of the amine/aldehyde functional units, (**1.5:1** and **2:1**), and keeping constant the amount of drug (1.5 mg), two formulations coded **D1.5** and **D2** were prepared. Thus **D1.5** formulation was prepared by crosslinking of 43.4 mg chitosan with 17.1 mg salicylaldehyde mixed with 1.5 mg DCF, and **D2** was prepared by crosslinking 46.71 mg chitosan with 13.79 mg salicylaldehyde mixed with 1.5 mg DCF.

### 2.3. Methods

The corresponding **xerogels** of the formulations were achieved after they have been lyophilized using a Labconco FreeZone Freeze Dry System equipment, for 24 h at −54 °C and 1.512mbar.

The morphology of the formulations was investigated on the corresponding xerogels, using a field emission **Scanning Electron Microscope (SEM)** EDAX – Quanta 200 at accelerated electron energy of 20 KeV.

The supramolecular ordering of the formulations was investigated on the corresponding xerogels by **polarized light microscopy (POM)** with a Leica DM 2500 microscope.

### 2.4. The in vitro DCF release protocol

The *in vitro* drug release has been monitored over 10 days, simulating the *in vivo* physiological conditions, by using phosphate buffer solution (PBS) of pH 7.4 and keeping a constant temperature at 37 °C on the entire investigation period. The formulations of similar amounts (62 mg) were prepared as pills by pressing into a hydraulic press (2 N/m^2^). The pills were dipped into vials containing 10 mL of PBS. At fixed intervals, 2 mL aliquots were withdrawn and replaced with 2 mL fresh buffer. The supernatant samples were collected and the *DCF* amount was determined by quantitative absorption spectroscopy, recording the characteristic absorption band at 275 nm, and fitting its absorbance on a predetermined calibration curve (Iftime et al., 2020; Dragan & Cocarta, 2016). The cumulative release of the *DCF* was estimated from the Lambert-Beer law. The UV-Vis spectra were recorded on an UV-visible spectrophotometer (Perkin Elmer, Lambda 10).

### 2.5. Theoretical consideration

In order to assess the mechanism of the *DCF* release from the formulations on the two stages, the data were fitted on the following models (Ritger & Peppas, 1987; Masaro & Zhu, 1999; Siepmann & Peppas, 2011):

- Zero order model: ***Q_t_ = k_o_ · t****_,_* where Q_t_ is the quantity of DCF dissolved in the time *t* and K_0_ is the zero order release constant.
- First order model: ***logQ_t_ = k · t/2.303***, where Q_t_ is the amount of DCF released in the time *t* and K is the first order release constant.
- Korsmeyer-Peppas model: ***M_t_/M_∞_ = k · t^n^***, where M_t_/M_∞_ is the fraction of DCF released at the time *t*, K is the release rate constant and n is the release exponent.
- Higuchi model: ***Q_t_ = k_H_ · t^1/2^***, where Q_t_ is the amount of DCF released in the time *t* and K_H_ is the Higuchi dissolution constant.
- Hixson-Crowell model: ***Wo^1/3^-Wt^1/3^ = k · t***, where W_0_ is the initial amount of DCF in formulations, W_t_ is the remaining amount of DCF in formulation at time *t* and K is a constant.

#### 2.5.1. Short reminder on non – differentiability calibrated on drug release process

Considering that the dynamics of the polymer-drug structural units take place on continuous but non – differentiable curves (multifractal curves), these dynamics will be described through the scale covariance derivative (for details see Merches & Agop, 2016):

(1) $$\frac{\hat{d}}{dt}=\partial_{t}+{\hat{V}}^{l}\partial_{l}+\frac{1}{4}{\left(dt\right)}^{[\frac{2}{f(\alpha )}]-1}D^{lp}\partial_{l}\partial_{p},$$

Where

(2) $$\begin{array}{c} \begin{array}{c} {\hat{V}}^{l}=V_{D}^{l}-V_{F}^{l}\\ D^{lp}=d^{lp}-i{\hat{d}}^{lp}\\ d^{lp}=\lambda_{+}^{l}\lambda_{+}^{p}-\lambda_{-}^{l}\lambda_{-}^{p} \end{array}\\ {\hat{d}}^{lp}=\lambda_{+}^{l}\lambda_{+}^{p}+\lambda_{-}^{l}\lambda_{-}^{p}\\ \partial_{t}=\frac{\partial }{\partial t},\;\partial_{l}=\frac{\partial }{\partial x^{l}},\;\partial_{l}\partial_{p}=\frac{\partial }{\partial x^{l}}\frac{\partial }{\partial x^{p}},\;i=\sqrt{-1},\;l,p=1,2,3 \end{array}$$

In the above – written relations, $x^{l}$ is the fractal spatial coordinate, t is the non – fractal time having the role of an affine parameter of the motion curves, ${\hat{V}}^{l}$ is the complex velocity, $V_{D}^{l}$ is the differential velocity independent on the scale resolution dt, $V_{F}^{l}$ is the non – differentiable velocity dependent on the scale resolution, $D^{lp}$ is the constant tensor associated with the differentiable – non – differentiable transition, $\lambda_{+}^{l}(\lambda_{+}^{p})$ is the constant vector associated with the backward differentiable – non – differentiable drug release $\lambda_{-}^{l}(\lambda_{-}^{p})$ is the constant vector associated with the forward differentiable – non – differentiable drug release processes, f(α) is the singularity spectrum of order α of the fractal dimension *D_F_* and α is the singularity index. There are many modes, and thus a varied selection of definitions of fractal dimensions: the fractal dimension in the sense of Kolmogorov, the fractal dimension in the sense of Hausdorff – Besikovitch etc. (Mandelbrot, 1982; Jackson, 1993; Cristescu, 2008). Selecting one of these definitions and operating it in the drug release dynamics, the value of the fractal dimension must be constant and arbitrary for the entirety of the compartmental analysis: for example, it is regularly found $D_{F}<2$ for drug release correlative processes, $D_{F}>2$ for drug release non – correlative processes, etc. In such a conjecture, through (3) it is possible to identify not only the ‘areas’ of the drug release dynamics that are characterized by a certain fractal dimension (mono-fractal drug release dynamics), but also the number of ‘areas’ whose fractal dimensions are situated in an interval of values (multifractal drug release dynamics). More than that, through the singularity spectrum f(α) it is possible to identify classes of universality in the drug release dynamics laws, even when regular or strange attractors have different aspects.

If the drug release dynamics are described through Markov – type stochastic processes (Mandelbrot, 1982; Jackson, 1993; Cristescu, 2008):

(3) $$\lambda_{+}^{i}\lambda_{+}^{l}=\lambda_{-}^{i}\lambda_{-}^{l}=2\lambda \delta^{il}$$

and for

(4) $$f(\alpha )\equiv D_{F}$$

where λ is a specific coefficient associated to the fractal – non – fractal scale transition and $\delta^{il}$ is Kronecker’s pseudo – tensor, the scale covariant derivative becomes:

(5) $$\frac{d}{dt}=\partial_{t}+{\hat{V}}^{l}\partial_{l}-i\lambda {\left(dt\right)}^{(\frac{2}{D_{F}})-1}\partial_{l}\partial^{l}$$

In the context for drug release dynamics described by means of motions on Peano – type curves, which implies $D_{F}=2,$ the scale covariant derivative (5) takes the standard form from the Scale Relativity Theory (Nottale, 2011):

(6) $$\frac{d}{dt}=\partial_{t}+{\hat{V}}^{l}\partial_{l}-iD\partial_{l}\partial^{l}$$

where λ≡D is the diffusion coefficient associated to fractal – non – fractal scale transition. Therefore, this model, generalizes all the results of Nottale’s theory (i.e., Scale Relativity Theory) (Nottale, 2011). Now, accepting the functionality of the scale covariance principle, i.e., applying the operator (1) to the complex velocity fields (2), in the absence of any external constraint, the motion equations of the polymer-drug structural units dynamics (i.e., the geodesics equation on multifractal space) takes the following form:

(7) $$\frac{d{\hat{V}}^{i}}{dt}=\partial_{t}{\hat{V}}^{i}+{\hat{V}}^{l}\partial_{l}{\hat{V}}^{i}+\frac{1}{4}{\left(dt\right)}^{[\frac{2}{f(\alpha )}]-1}D^{lk}\partial_{l}\partial_{k}{\hat{V}}^{i}=0,$$

This means that the multifractal acceleration, $\partial_{t}{\hat{V}}^{i},$ the multifractal convection, ${\hat{V}}^{l}\partial_{l}{\hat{V}}^{i}$ and the multifractal dissipation $D^{lk}\partial_{l}\partial_{k}{\hat{V}}^{i}$ make their balance in every point of any multifractal curve (Irimiciuc et al., 2018) of the polymer drug structural units dynamics. Particularly, for (3) and (4), the motion Equation (7) becomes:

(8) $$\frac{\hat{d}{\hat{V}}^{i}}{dt}=\partial_{t}{\hat{V}}^{i}+{\hat{V}}^{l}\partial_{l}{\hat{V}}^{i}-i\lambda {\left(dt\right)}^{[\frac{2}{D_{F}}]-1}\partial_{l}\partial^{l}{\hat{V}}^{i}=0$$

Now, separating the polymer- drug structural units dynamics on scale resolutions (the differentiable and non – differentiable scale resolutions), (7) becomes:

(9) $$\begin{array}{c} \partial_{t}V_{D}^{i}+V_{D}^{l}\partial_{l}V_{D}^{i}-V_{F}^{l}\partial_{l}V_{F}^{i}+\frac{1}{4}{\left(dt\right)}^{[\frac{2}{f(\alpha )}]-1}D^{lk}\partial_{l}\partial_{k}V_{D}^{i}=0\\ \partial_{t}V_{F}^{i}+V_{F}^{l}\partial_{l}V_{D}^{i}+V_{D}^{l}\partial_{l}V_{F}^{i}-\frac{1}{4}{\left(dt\right)}^{[\frac{2}{f(\alpha )}]-1}D^{lk}\partial_{l}\partial_{k}V_{F}^{i}=0, \end{array}$$

while (8) takes the form:

(10) $$\begin{array}{c} \partial_{t}V_{D}^{i}+V_{D}^{l}\partial_{l}V_{D}^{i}-[V_{F}^{l}+\lambda {\left(dt\right)}^{[\frac{2}{f(\alpha )}]-1}\partial^{l}]\partial_{l}V_{F}^{i}=0\\ \partial_{t}V_{F}^{i}+V_{D}^{l}\partial_{l}V_{F}^{i}+[V_{F}^{l}+\lambda {\left(dt\right)}^{[\frac{2}{f(\alpha )}]-1}\partial^{l}]\partial_{l}V_{D}^{i}=0, \end{array}$$

For irrotational motions of the polymer-drug structural units, the complex velocity fields (5) take the form:

(11) $${\hat{V}}^{i}=-2i\lambda (dt{)}^{[\frac{2}{f(\alpha )}]-1}\partial^{i}\ln\Psi$$

where Ψ is the states function. From here,

(12) $$\Psi =\sqrt{\rho }e^{is},$$

where $\sqrt{\rho }$ is the amplitude and s is the phase, the complex velocity fields (11) become explicit:

(13) $${\hat{V}}^{i}=2\lambda (dt{)}^{[\frac{2}{f(\alpha )}]-1}\partial^{i}s-i\lambda (dt{)}^{[\frac{2}{f(\alpha )}]-1}\partial^{i}\ln\rho$$

which enable the definition of the velocity fields:

(14) $$V_{D}^{i}=2\lambda (dt{)}^{[\frac{2}{f(\alpha )}]-1}\partial^{i}s$$

(15) $$V_{F}^{i}=i\lambda (dt{)}^{[\frac{2}{f(\alpha )}]-1}\partial^{i}\ln\rho$$

By (12), (14) and (15) and using the mathematical procedures from (Merches & Agop, 2016; Agop & Paun, 2017), the geodesics Equation (13) reduces to the multifractal hydrodynamic – type equations:

(16) $$\partial_{t}V_{D}^{i}+V_{D}^{l}\partial_{l}V_{D}^{i}=-\partial^{i}Q$$

(17) $$\partial_{t}\rho +\partial_{l}(\rho V_{D}^{l})=0$$

with Q the specific multifractal potential:

(18) $$Q=-2\lambda^{2}(dt{)}^{[\frac{4}{f(\alpha )}]-2}\frac{\partial^{l}\partial_{l}\sqrt{\rho }}{\sqrt{\rho }}=-V_{F}^{i}V_{F}^{i}-\frac{1}{2}\lambda (dt{)}^{[\frac{2}{f(\alpha )}]-1}\partial_{l}V_{F}^{l}$$

The Equation (16) corresponds to the specific momentum conservation law of multifractal type, while Equation (17) corresponds to the states density conservation law of multifractal type. The specific multifractal potential (18) implies the specific multifractal force:

(19) $$F^{i}=-\partial^{i}Q=-2\lambda^{2}(dt{)}^{[\frac{4}{f(\alpha )}]-2}\partial^{i}\frac{\partial^{l}\partial_{l}\sqrt{\rho }}{\sqrt{\rho }}$$

which is a measure of the multifractality of the motion curves.

#### 2.5.2. Synchronous drug release phenomena

Let it be considered the one – dimensional multifractal hydrodynamic – type Equations (16–18), in the form:

(20) $$\partial_{t}V_{D}+V_{D}\partial_{x}V_{D}=-\partial_{x}[-2\lambda (dt{)}^{[\frac{4}{f(\alpha )}]-2}\frac{\partial_{x}\partial_{x}\sqrt{\rho }}{\sqrt{\rho }}]$$

(21) $$\partial_{t}\rho +\partial_{x}(\rho V_{D})=0$$

These equations for the initial and boundary conditions:

(22) $$V_{D}(x,t=0)=V_{0},\;\rho (x,t=0)=\frac{1}{\sqrt{\pi }\alpha }\text{exp}\;[-{\left(\frac{x}{\alpha }\right)}^{2}]$$

(23) $$V_{D}(x=V_{0}t)=V_{0},\;\rho (x=-\infty ,t)=\rho (x=+\infty ,t)=0$$

with $V_{0}$ the initial velocity and α the parameter of Gaussian distribution of positions, using the mathematical procedures from (Merches & Agop, 2016; Agop & Paun, 2017), admit the solution:

(24) $$V_{D}(x,t,\sigma ,\alpha )=\frac{V_{0}\alpha^{2}+{\left(\frac{\sigma }{\alpha }\right)}^{2}xt}{\alpha^{2}+{\left(\frac{\sigma }{\alpha }\right)}^{2}t^{2}}$$

(25) $$\rho (x,t,\sigma ,\alpha )=\frac{\pi^{-1/2}}{{\left(\alpha^{2}+{\left(\frac{\sigma }{\alpha }\right)}^{2}t^{2}\right)}^{1/2}}e\mathrm{xp}[-\frac{{\left(x-V_{0}t\right)}^{2}}{\alpha^{2}+{\left(\frac{\sigma }{\alpha }\right)}^{2}t^{2}}]$$

with

(26) $$\sigma =\lambda (dt{)}^{[\frac{2}{f(\alpha )}]-1}$$

the multifractal degree. From here, through (15) the non – differentiable velocity $V_{F}$ takes the form:

(27) $$V_{F}(x,t,\sigma ,\alpha )=\sigma \frac{\left(x-V_{0}t\right)}{\alpha^{2}+{\left(\frac{\sigma }{\alpha }\right)}^{2}t^{2}}$$

Introducing the non – dimensional variables:

(28) $$\xi =\frac{x}{V_{0}\tau_{0}},\;\eta =\frac{t}{\tau_{0}}$$

and non – dimensional parameters

(29) $$\mu =\frac{\sigma \tau_{0}}{\alpha^{2}},\;\phi =\frac{\alpha }{{V_{0}\tau }_{0}}$$

with $\tau_{0}$ the specific time, (24), (25) and (27) become:

(30) $$V\equiv V_{D}(\xi ,\eta ,\mu )=\frac{V_{D}(x,t,\sigma ,\alpha )}{V_{0}}=\frac{1+\mu^{2}\xi \eta }{1+\mu^{2}\eta^{2}}$$

(31) $$\rho (\xi ,\eta ,\mu ,\phi )=\pi^{\frac{1}{2}}\alpha \rho (x,t,\sigma ,\alpha )={\left(1+\mu^{2}\eta^{2}\right)}^{-\frac{1}{2}}\;\text{exp}\;[-\frac{{\left(\xi -\eta \right)}^{2}}{\phi^{2}(1+\mu^{2}\eta^{2})}]$$

(32) $$U\equiv V_{F}(\xi ,\eta ,\mu )=\frac{V_{F}(x,t,\sigma ,\alpha )}{V_{0}}=\mu \frac{\left(\xi -\eta \right)}{1+\mu^{2}\eta^{2}}$$

Now taking out the quadratic term in η between (30) and (32), it results that for ξ=const. the ratio $\frac{V}{U}$ is homographic dependent of ξ by the form:

(33) $$\frac{U}{V}=\frac{\mu (\xi -\eta )}{1+\mu^{2}\xi \eta }$$

From here, the condition (dynamical simultaneity):

(34) $$d(\frac{U}{V})=0⟺V=\mathrm{constU}$$

(i.e., the extension of the first principle of Newton to any scale resolution, or equivalently, ‘synchronizations’ of drug release dynamics at differentiable scale with drug release dynamics at non – differentiable scale), implies correlations between phase and amplitude of the shape function, by the form:

(35) $$\ln\rho =\rho_{0}\;\text{exp}\;[\mathrm{const}(s-s_{0})]$$

where $\rho_{0}$ and $s_{0}$ are integration constants. Thus, it is stated that various ‘mechanisms’ involved in the drug release process can be mimed through period doubling, quasi – periodicity, intermittences etc. (for details see Ailincai et al., 2020).

**Figure 1.**
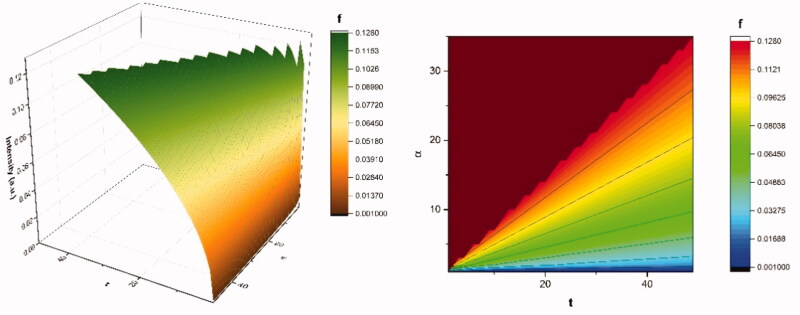
3 D (left-side) and contour plot (right-side) representations of our multifractal function (Equation (38)) use for drug release mechanism analysis.

Because through the restriction (34) given, for example, by V=−U, the multifractal type conservation laws (20) and (21) take the form of the multifractal type ‘diffusion’ equation:

(36) $$\partial_{t}\rho =\lambda (dt{)}^{[\frac{2}{f(\alpha )}]-1}\partial_{l}\partial^{l}\rho =\sigma \partial_{l}\partial^{l}\rho$$

it results that these ‘mechanisms’ ‘manifest’/are ‘perceived’ as diffusions at various scale resolutions in a multifractal space (fickian – type diffusion, non – fickian – type diffusion etc.) To explain such a situation: the one – dimensional drug diffusion of multifractal type from a controlled – release polymeric system with the form of a plane shut, of thickness δ. If drug release of multifractal type occurs under perfect sink condition, the following initial and boundary conditions can be assumed:

(37) $$\begin{array}{c} t=0,\;\;-\;\frac{\alpha }{2}<x<\frac{\alpha }{2},\;\;\rho =\rho_{0}\\ t\;>\;0,\;\;x=\pm \frac{\alpha }{2},\;\;\rho =\rho_{1} \end{array}$$

where $\rho_{0}$ is the initial drug states density of the multifractal type in the ‘device’ of multifractal type and $\rho_{1}$ is the drug states density at the ‘polymer – fluid’ interface of multifractal type. This solution equation under these conditions can take the following form (for details in the classical case see Crank, 1965). In Figure 1 there are represented the

(38) $$f=\frac{\rho_{t}}{\rho_{\infty }}=2{\left(\frac{\sigma t}{\delta^{2}}\right)}^{\frac{1}{2}}=\{\pi^{-1/2}+\sum_{n=1}^{\infty }{\left(-1\right)}^{n}e\mathrm{rfc}[\frac{n\delta }{2{\left(\sigma t\right)}^{\frac{1}{2}}}]\}$$

An accurate expression can be obtained for small values of t since the second term of (38) disappears and then it becomes:

(39) $$\frac{\rho_{t}}{\rho_{\infty }}=2{\left(\frac{\sigma t}{\delta^{2}}\right)}^{\frac{1}{2}}=\mathrm{const}{\left(t\right)}^{\frac{1}{2}}$$

In such a context, $\frac{\rho_{t}}{\rho_{\infty }}$ can be assimilated to the fraction of dissolved drug i.e., $\frac{M_{t}}{M_{\infty }}\equiv \frac{\rho_{t}}{\rho_{\infty }},$ where $M_{t}$ is the amount of drug dissolved in time t and $M_{\infty }$ is the total amount of time dissolved when the pharmaceutical dosage form is exhausted.

## 3. Results and discussion

**Figure 2.**
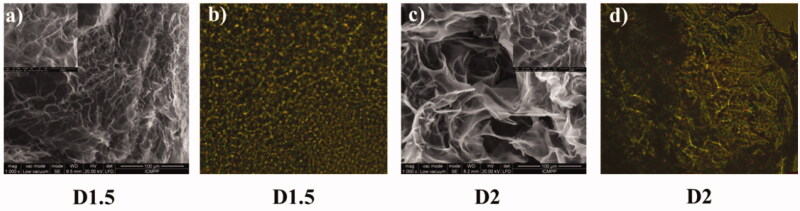
SEM and POM images of the **D1.5** and **D2** formulations.

In view of modeling the drug release characteristic, two formulations based on chitosan, salicylaldehyde and *DCF* (noted **D1.5 and D2**) were prepared applying the procedure of the *in situ* hydrogelation described in Section Materials and methods. The formulations have different crosslinking density (NH_2_/CHO = 1.5 or 2, respectively) and the same amount of encapsulated drug. As the formulation impacts the drug delivery by both, size and distribution of the pores of hydrogel on a hand, and size and distribution of the drug crystals on the other hand, the formulation morphology was evaluated by scanning electron microscopy (SEM). As can be seen in Figure 2(a,c) the samples revealed a porous morphology, with interconnected pores, without visible drug crystals, indicating that the DCF was encapsulated into the pore walls at submicrometric level. This observation was further confirmed by polarized light microscopy (POM) which displayed strong banded birefringence, characteristic for layered ordered phases (Marin et al., 2009) and no crystals of drug into the pores walls (Figure 2(b,d)). This reinforces the idea that the DCF drug is intimately mixed with the chitosan-based matrix, probably by intermolecular forces, forming ‘fractal’ drug-polymer structural units. Starting from this hypothesis the DCF release can be seen as a progressive release of the fractals of different size.

**Figure 3.**
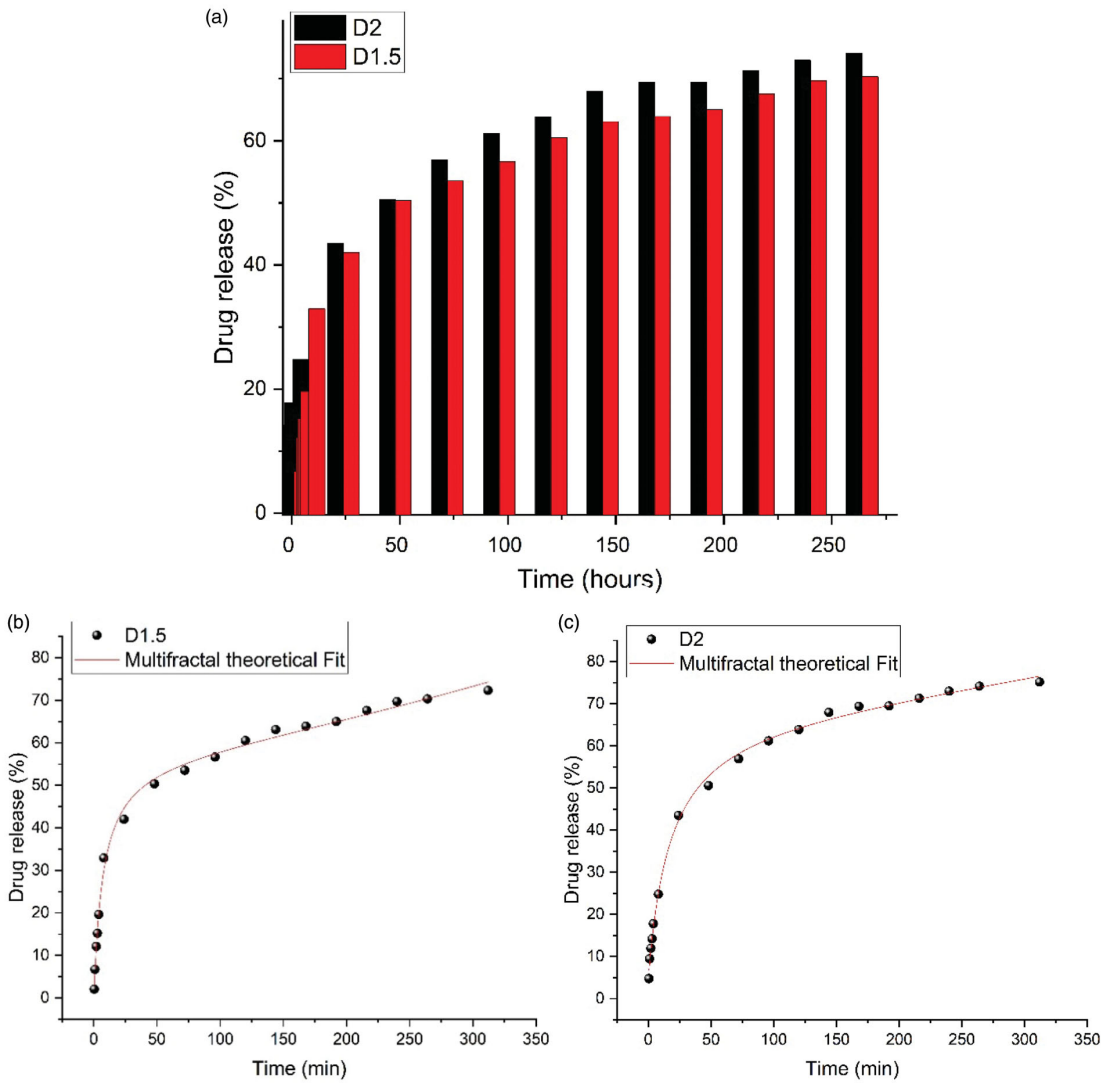
Graphical representation of the drug release from **D1.5** and **D2** formulations (a) and comparison with the multifractal theoretical model (b,c).

The *in vitro* release investigation revealed a progressive release of the DCF in two well delimited stages, as can be seen in the graphical representation depicted in Figure 3(a): a burst release during 8 hours in the *first stage*, and a slower, continuous release during 10 days in the *second stage*. The two stages look slightly different for the two samples, reflecting the influence of the crosslinking ratio and the hydrogelation speed. Thus, in the *first stage* **D1.5** formulation released 33% DCF, while **D2** only 25%. On the contrary, in the *second stage*, a more rapid release was noted for the **D2** formulation. Both samples reached a total drug release of 70% after 10 days. In the light of the fractal formation hypothesis, it can be appreciated that in the case of **D1.5** with a faster hydrogelation rate of the more viscous system, the drug was most probably encapsulated as bigger crystals (Ailincai et al., 2018), forming a larger fraction of fractals richer in DCF, more prone for dissolution in the release medium in the *first stage*. This can explain their faster dissolution in the *first stage*, followed by a sustained release over 10 days, of the larger remnant fraction of the fractals scarcer in DCF. In the case of formulation with lower crosslinking ratio, **D2**, the slower hydrogelation rate allowed a finer distribution of the *DCF* into the hydrogels matrix forming a larger fraction of scarcer DCF fractals, slowing down the release in the *first stage* and supporting a sustained release in the *second stage*. In Figure 3(b,c) we have compared the experimental date for both the **D1.5** and **D2** with the predictions made by our multifractal drug release model presented in the previous section. The calibration of the model onto the experimental particularities and the biochemistry of the drug-polymer matrix are performed by fitting the empirical data with the multifractal function. We do notice a good correlation between the theoretical simulated prediction and the empirical data.

**Figure 4.**
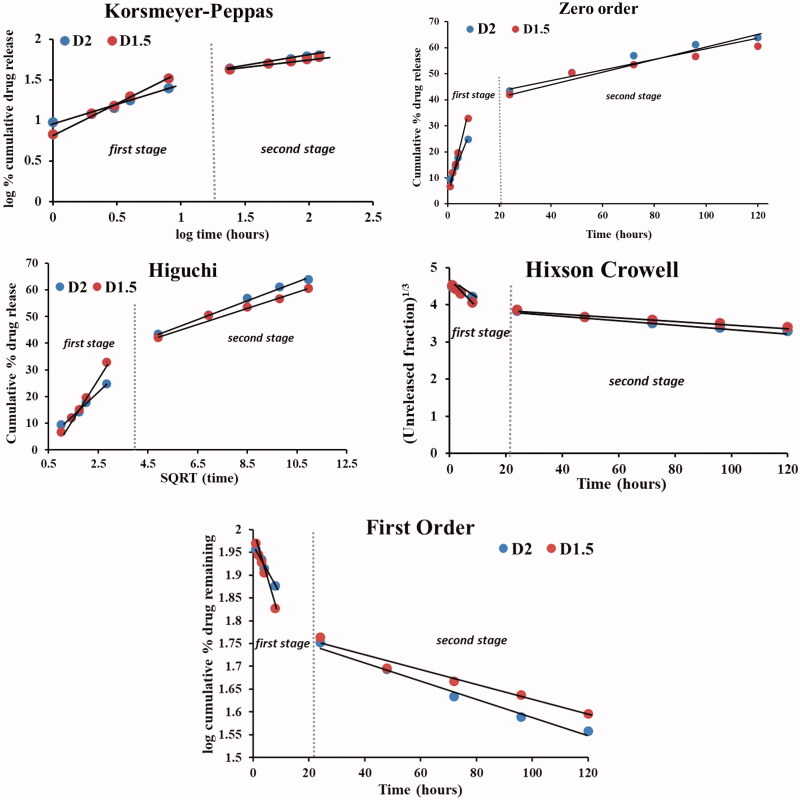
Linear forms of the *Korsmayer-Peppas, Zero Order*, *Higuchi, Hixson-Crowell, First order* models applied for the release of *DCF* from **D1.5** and **D2** on the *first* and *second stage*.

The kinetics release mechanism of the *DCF* drug from **D1.5** and **D2** samples was assessed by fitting the *in vitro* release data on the mathematical equations of the *Korsmeyer-Peppas, Zero order, First order, Higuchi and Hixson-Crowell*, on each of the two stages (Figure 4, Table 1). As it can be seen in Figure 4(a,b), all *in vitro* release data proved a good fitting in the *first stage* (Figure 4(a)) and *second stage* (Figure 4(b)) on the all five mathematical models. This indicates that the DCF release mechanism is controlled by both dissolution velocity and diffusion through the hydrogel network. This conclusion fits very well with the findings of the theoretical mathematical model, converging to the conclusion of a multifractal composition of the drug release systems which determines a progressive release of the dominant fractal population.

**Table 1.** Results of the fitting of the release curves on the five different mathematical models.

Model	*Zero Order*		*First Order*		*Higuchi*		*Korsmeyer-Peppas*			*Hixson-Crowell*	
Code	R^2^	k_0_	R^2^	k	R^2^	k_H_	R^2^	k	*n*	R^2^	k
CSD1.5*	0.994	3.7	0.998	0.05	0.994	14.2	0.997	0.15	0.75	0.997	−0.07
CSD2*	0.985	2.2	0.990	0.03	0.989	8.6	0.982	0.11	0.47	0.988	−0.04
CSD1.5**	0.950	0.18	0.970	0.004	0.983	2.9	0.991	0.05	0.22	0.970	−0.005
CSD2**	0.970	0.21	0.985	0.005	0.994	3.5	0.997	0.05	0.25	0.980	−0.006

*First stage =1–8h; **Second stage = 1–5 days.

Some correlations can be made between the multifractal and classical approaches; however these need to be understood as mostly qualitatively, considering the early stage of development for our multifractal model. The multifractal model can offer as output parameters fractalization dimension or scale resolution which can potentially be correlated with the transport constants. If we implement the fractal analysis in a sequential manner, as classical models do, we obtain for the first stage a fractalization degree between 2.4 (D2) and 1.6 (D1.5), while for the second stage we get value about one order of magnitude lower 0.7 (D2) and 0.4 (D1.5). This is in line with the differences seen in the constant transports given by Korsmeyer-Peppas or Hixson-Crowell models. Therefore, the transport constants can be corelated with the fractality degree of the drug release process. There is also a clear difference between the fractalization degrees for the D2 and D1.5 which is a direct reflection of the increased number of interactions in the drug release exchange.

## 4. Conclusions

Two diclofenac release systems based on a chitosan were prepared by an *in situ* hydrogelation process with different amounts of salicylaldehyde crosslinker, leading to systems with different crosslinking degree. The *in vitro* release and fitting on traditional models revealed a prolonged drug delivery mainly controlled by dissolution velocity and diffusion through the matrix. A different drug release rate noticed in the first release stage was attributed to the formation of polymer-drug structural units of different size, controlled by the system viscosity during the hydrogelation step. This indicated the formation of a multifractal system during the hydrogelation which controls the rate of drug release.

A theoretical model in a multi fractal paradigm was developed for understanding the drug release dynamics, considering that these behaviors are described by continuous but nondifferentiable curves. In such a context the irrotational type dynamic of the polymer drug structural units implies the functionality of a multifractal type hydrodynamic formalism. For the unidimensional case of this multifractal type hydrodynamic formalism, it was seen that ratio between the differentiable velocity and the nondifferentiable one for a certain distance depends on a homographic manner on time. The conditions for the simultaneous dynamics imply the synchronization of the drug release mechanisms at the two scale resolutions, expressed through diffusion functions of multifractal type (the diffusion process depends on the scale resolutions).
